# The ubiquitin E3 ligase ITCH enhances breast tumor progression by inhibiting the Hippo tumor suppressor pathway

**DOI:** 10.18632/oncotarget.2540

**Published:** 2014-10-11

**Authors:** Zaidoun Salah, Ella Itzhaki, Rami I Aqeilan

**Affiliations:** ^1^ The Lautenberg Center for Immunology and Cancer Research, IMRIC, Hebrew University-Hadassah Medical School, Jerusalem, Israel; ^2^ Al Quds-Bard College, Al-Quds University, Abu Dies, East Jerusalem, Palestine; ^3^ Department of Molecular Virology, Immunology and Medical Genetics, The Ohio State University Wexner Medical Center, Columbus, OH 43210, USA

**Keywords:** Hippo pathway, ITCH, YAP, breast cancer, metastasis

## Abstract

The Hippo kinase pathway is emerging as a conserved signaling pathway that is essential for organ growth and tumorigenesis. Recently, we reported that the ubiquitin E3 ligase ITCH negatively regulates LATS1, thereby increasing YAP activity, which leads to increased cell proliferation and decreased apoptosis. Here, we investigated the role of ITCH in breast tumorigenesis. In particular, we show that ITCH enhances epithelial-to-mesenchymal transition (EMT) through boosting YAP oncogenic function. By contrast, a point mutation in the catalytic domain or WW1 domain of ITCH abolished its EMT-mediated effects. Furthermore, while overexpression of ITCH expression in breast cells is associated with increased incidence of mammary tumor formation and progression, its knockdown inhibited breast cancer cell tumorigenicity and metastasis. Importantly, YAP knockdown was able to attenuate ITCH pro-tumorigenic functions. Lastly, we found that ITCH expression is significantly upregulated in invasive and metastatic breast cancer cases and is associated with worse survival. Together, our results reveal that ITCH pro-tumorigenic functions in breast cancer are mediated, at least in part, through inactivation of the Hippo tumor suppressor pathway.

## INTRODUCTION

The Hippo kinase pathway is emerging as a conserved signaling pathway that controls cell proliferation, apoptosis, contact inhibition, cell migration, cell differentiation, stem cell self-renewal, genetic stability, and epithelial-to-mesenchymal transition (EMT). Because it plays such versatile roles in cellular and tissue homeostasis, the Hippo signaling pathway appears to be essential for organ growth and homeostasis and its impairment leads to tissue overgrowth and tumor development and metastasis of different cancer types [1–3]

The core of the Hippo pathway is composed of a kinase cascade that includes MST1/2 serine/threonine kinase (ortholog of Hpo in *D. melanogaster*), WW45 scaffold protein (Sav), MOB (Mats) and LATS1/2 kinases (Wts). Activation of the core cascade leads to phosphorylation of YAP [4–6] (Yki in flies) and TAZ [7]. Phosphorylation of these oncoproteins leads to a complex formation between YAP or TAZ and 14-3-3 or to their β-TRCP-dependent proteasomal degradation [6, 8]. Both of these mechanisms prevent YAP and TAZ translocation to the nucleus and binding to TEAD transcription factors, thereby inhibiting transcription of downstream target genes implicated in proliferation, anti-apoptosis and EMT [9]. The Hippo pathway is activated mainly through the activation of its kinase cascade, which can be triggered by different upstream proteins such as Kibra–Expanded–Merlin complex in *D. melanogaster*; and KIBRA, WILLIN and NF2, in mammals [1, 2, 10]. Other proteins capable of activating the core cascade of the Hippo pathway include the apicobasal cell polarity (ABCP) proteins, including mammalian Scribble (SCRIB) [1, 11, 12] and G-protein coupled receptors that are capable of activating LATS1/2 independent of MST1/2 [13].

Although the main mechanism in the regulation of the Hippo pathway is through activating its core kinases, other independent factors are also involved in its regulation. These mechanisms include sequestering the downstream effectors YAP and TAZ in the cytoplasm by different proteins such as the cell junction proteins angiomotin (AMOT) [14–16] and α-catenin [17]. Another mechanism that was shown to regulate the Hippo pathway function is the regulation of the abundance of numerous Hippo pathway components by various E3 ligases [18–20]. Indeed, two reports have recently shown that E3 ligase ITCH ubiquitinates LATS1 and mediates its proteasomal degradation [21]. This was accompanied by YAP accumulation and translocation into the nucleus thus phenocopying YAP activation [21]. ITCH is an ubiquitin E3 ligase that belongs to the NEDD4-like family of E3 ubiquitin ligases. ITCH contains 4 WW domains, known to associate with PPxY containing targets, conferring substrate specificity, and a HECT-type ligase domain providing the catalytic E3 activity [22]. ITCH was originally identified as a gene disrupted in the non-agouti–lethal 18H or *Itchy* mice that suffer from severe immune and inflammatory defects [23, 24]. A number of ITCH substrates that have been implicated in tumorogenesis and chemosensitivity have been identified, including c-Jun [25, 26], p73 [27], p63 [28], and ErbB4 [29].

In the current work, we dissected the role of ITCH in breast tumorigenesis. In particular, we show that ITCH enhances EMT, mammary tumor formation and metastasis through boosting YAP oncogenic function. Furthermore, ITCH knockdown inhibits breast cancer cell tumorigenicity and invasiveness, both *in vitro* and *in vivo*. Importantly, we found that ITCH levels are significantly increased in invasive and metastatic breast cancer samples.

## RESULTS

### ITCH overexpression induces a tumorigenic phenotype in MCF10A

**Figure 1:**
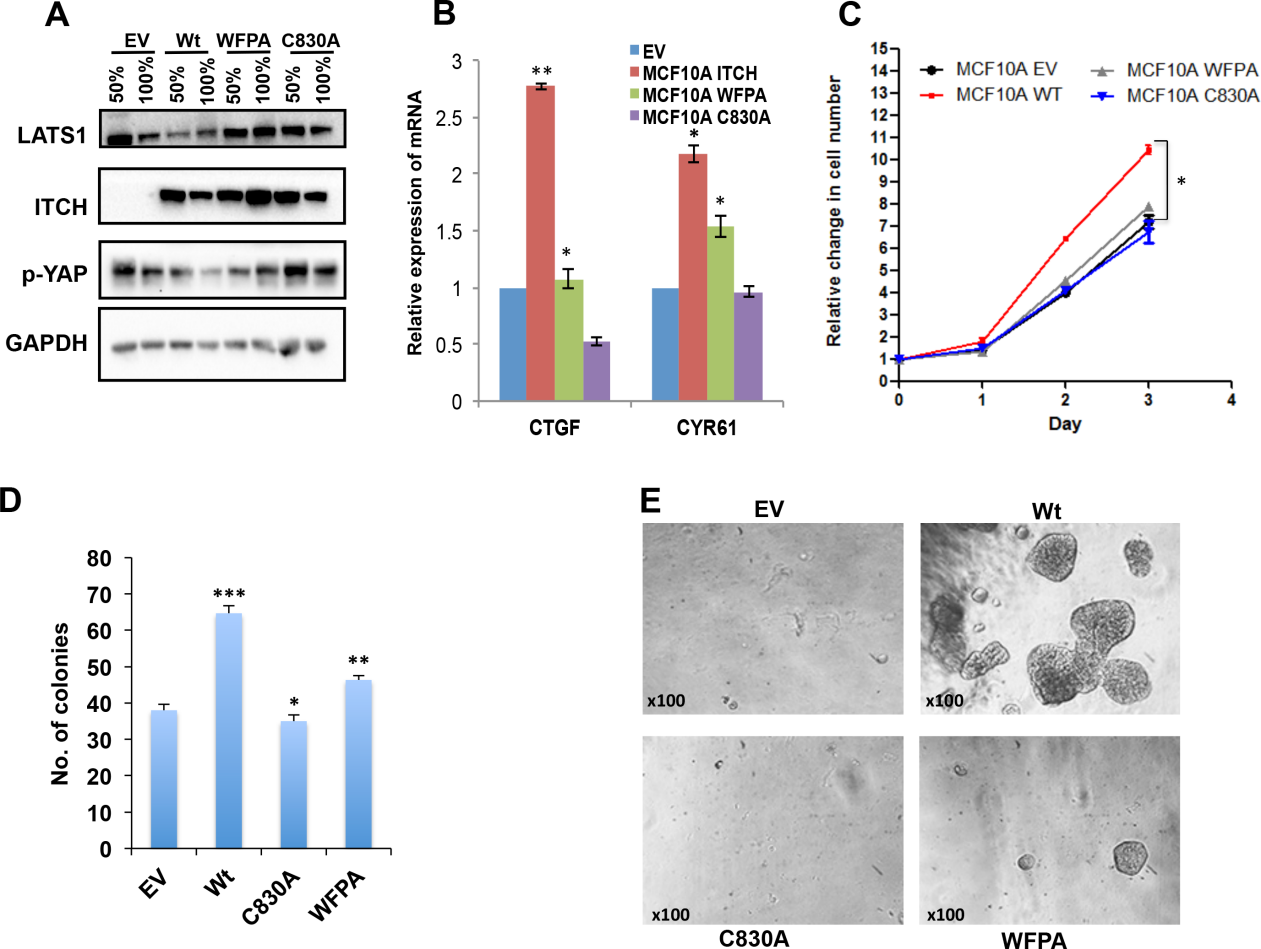
Effect of ITCH constructs overexpression in MCF10A cells **(A)** Immunoblot analysis showing the effect of overexpressing different ITCH constructs in MCF10A cells on LATS1 and pYAP levels upon 50% and 100% cell culture confluency. **(B)** Real time PCR showing the effect of different ITCH constructs on YAP target genes in 50% cell confluent culture. **(C)** XTT cell proliferation assay results showing the effect of ITCH manipulation on MCF10A growth rate. D & E. Colony formation **(D)** and 3D culture assay **(E)** experiments, respectively, showing the effect of ITCH manipulation on MCF10A cell survival. In all figures, error bars represent the standard deviation of at least three different biological experiments done in triplicates. The statistical significance was measured by calculating the p values for all experiments related to EV; * indicates *P* value <0.05, **P value < 0.01, ***P value <0.001.

We have recently shown that ITCH targets LATS1 for degradation and inhibits the function of the Hippo pathway [21]. To further investigate the role of ITCH in breast tumorigenesis and progression, we overexpressed either the wild type (wt), or the first WW domain (WFPA) or the catalytic domain (C830A) mutants ITCH in the immortalized mammary gland epithelial cells, MCF10A, using lentiviral vectors (Fig. 1A). To test the effect of the different ITCH constructs on endogenous LATS1 levels, we analyzed LATS1 protein levels in the different ITCH clones using immunoblot analysis. As shown in Fig 1A and Fig S1A, only wt ITCH was able to reduce endogenous LATS1 levels. Since LATS1 determines YAP phosphorylation levels and transactivation function, we analyzed ITCH clones for phospho-YAP (p-YAP) expression and YAP target genes, *CTGFβ* and *CYR61*. Only wt ITCH overexpression reduced p-YAP levels (Fig 1A) and increased YAP target gene expression (Fig 1B). Compared to C830A, which showed sometimes a dominant negative effect, a point mutation in the first WW domain of ITCH (ITCH-WFPA) displayed a milder effect on both YAP phosphorylation (Fig 1A, S1A) and YAP target gene expression (Fig 1B). To demonstrate the effect of the different ITCH constructs on breast cell phenotypes, we tested cell proliferation using XTT test, cell survival using colony formation assay or by culturing the cells in three dimensions (3D) in growth factor depleted Matrigel. Our results demonstrate that only wt ITCH was able to increase cell proliferation (Fig 1C) and cell survival (Fig 1D, E). These results suggest that ITCH enhances cell growth of MCF10A cells.

### ITCH supports a mesenchymal growth phenotype of MCF10A cells

**Figure 2:**
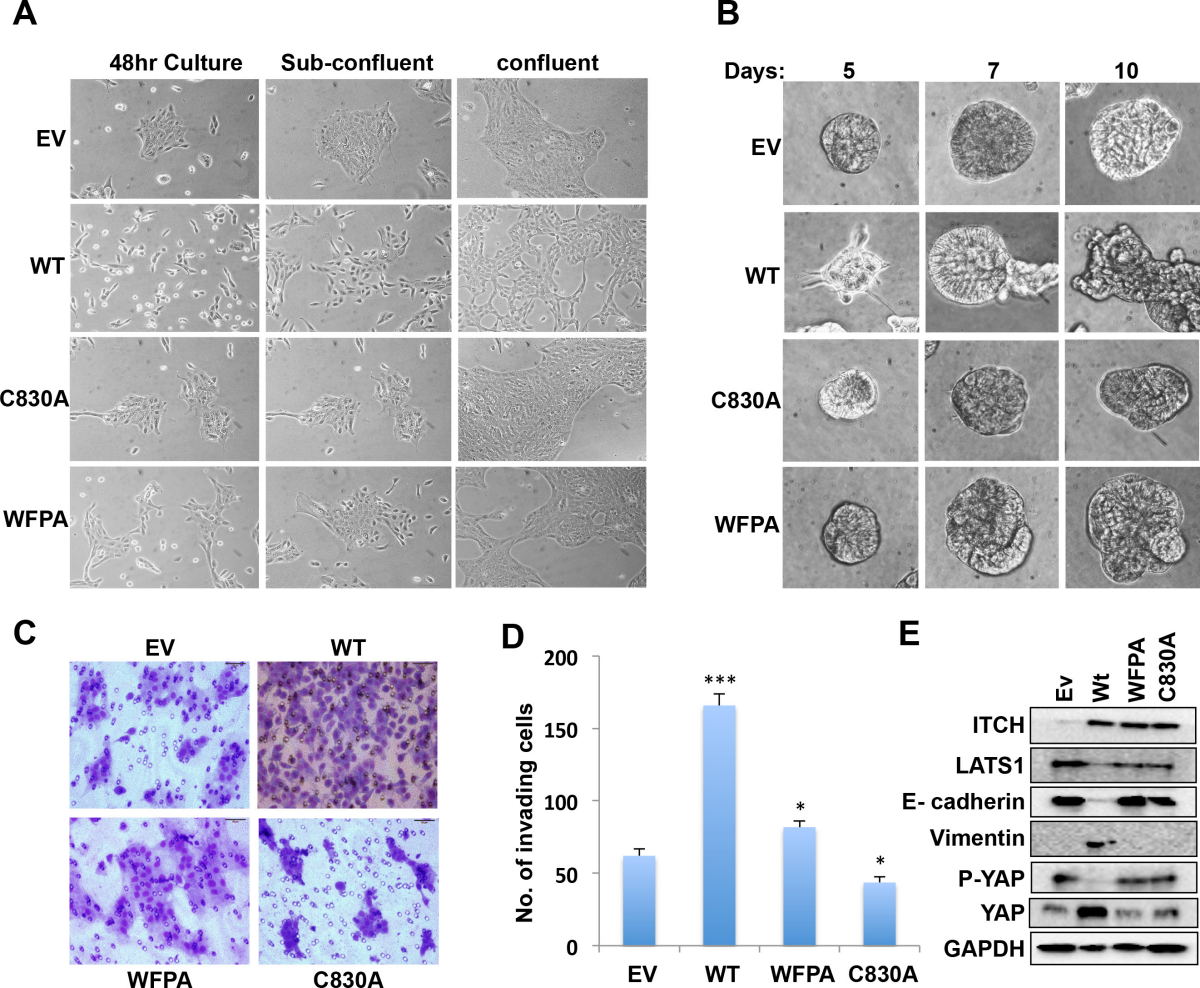
Effect of ITCH expression on EMT in MCF10A cells **(A)** Morphology changes of MCF10A cells expressing either empty vector (EV) or different ITCH forms when cultured in 2D. Images were obtained using inverted light microscope using 100X magnification. **(B)** Mammosphere formation in 3D Matrigel cell culture of MCF10A cells. Images were obtained using inverted light microscope using 200X magnification. **(C)** Representative images of Boyden Chamber Matrigel invasion assay of MCF10A cells. **(D)** Quantification of invading cells in C. **(E)** Immunoblot analysis showing the effect of overexpressing different ITCH constructs in MCF10A cells on EMT markers. In all figures, error bars represent the standard deviation of three different biological experiments done in triplicates. The statistical significance was measured by calculating the p values for all experiments related to EV; * indicates P value <0.05, **P value <0.01, ***P value <0.001.

The Hippo pathway controls proper tissue growth and homeostasis, and its deregulation promotes EMT of cultured cells, a hallmark of metastatic cancer cells. To examine the effect of ITCH on breast cancer progression in the context of the Hippo pathway, we tested the effect of different ITCH constructs on MCF10A cell morphology and mammosphere formation. When cultured in two dimensions (2D), control unmanipulated MCF10A cells tend to form well-circumscribed colonies even before filling the culture plates. In contrast, we found that overexpression of wt ITCH changes MCF10A cell morphology to a more mesenchymal phenotype precluding normal colony formation (Fig 2A). Both ITCH-WFPA and ITCH-C830A expression didn't affect cell morphology (Fig 2A).

When cultured in 3D, MCF10A cells are able to form very well organized mammospheres that recapitulate mammary cell growth *in vivo*. Our results revealed that overexpression of wt ITCH disrupt the formation of mammospheres, with mild versus no effect of WFPA and C830A ITCH constructs, respectively (Fig 2B). To further elucidate whether this change of breast cell morphology is accompanied with a more invasive potential, we tested the ability of the different MCF10A ITCH clones to invade Matrigel coated filters in a Boyden chamber Matrigel invasion assay. As shown in Fig 2C & D, wt ITCH expressing MCF10A cells displayed enhanced invasion ability relative to control or ITCH-WFPA cells. The ITCH-C830A expressing cells exhibited reduced invasion ability as compared to control cells again suggesting a dominant negative effect. To explain these pro-mesenchymal changes, we tested the effect of different ITCH constructs on levels of the epithelial marker E-cadherin and the mesenchymal marker Vimentin. First, we checked the expression levels of LATS1, p-YAP and total YAP to show that ITCH affects LATS1 expression and function. Our results show that only wt ITCH drastically decreases the expression of LATS1 and thus p-YAP while increasing total YAP levels indicating that only wt ITCH inhibits the Hippo pathway function (Fig 2E & S1B). Next we examined the effect of ITCH on EMT markers. While wt ITCH reduced E-cadherin and increased Vimentin levels, WFPA and C830A expressing cells were unable to do so (Fig 2E & S1B). These results suggest that ITCH enhances EMT and invasion potential of MCF10A cells.

### ITCH expression enhances MCF10A cell tumorigenesis *in vivo*

**Figure 3:**
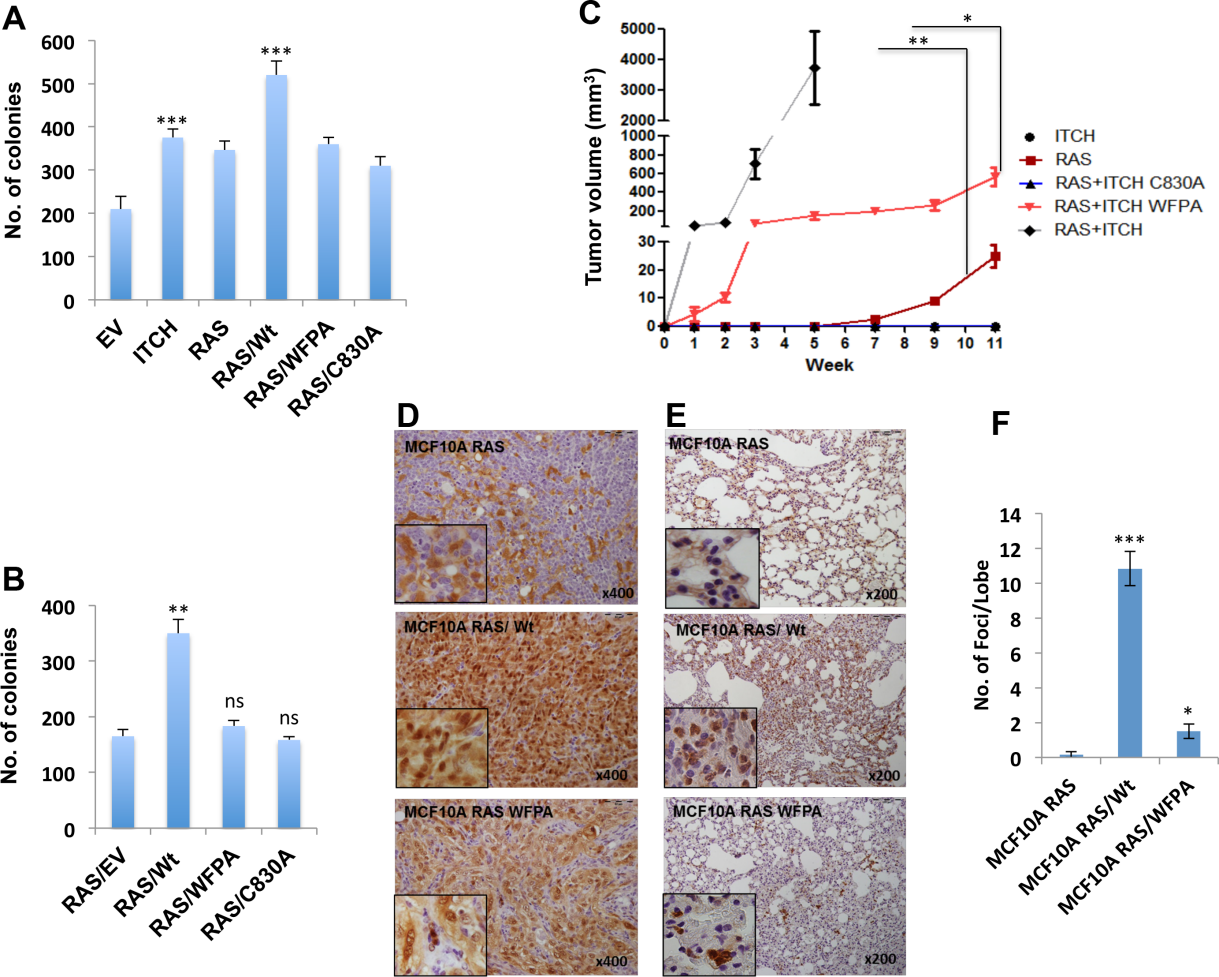
ITCH enhances tumorigenicity of H-RAS transformed MCF10A cells **(A)** Quantification of cell survival rates as assessed by colony formation assays. **(B)** Quantitative presentation of soft agar assays (Fig S2A) showing cell tumorigenic potential following the expression of the indicated proteins. **(C)** Tumor growth curve of MFP tumors in Nod-SCID mice injected with MCF10A overexpressing the indicated proteins. (n=6mice/group). **(D)** IHC staining for YAP in lungs obtained from mice described in C. Brown color indicates positive staining while blue color of the counter stain hematoxyline indicates a negative staining. **(E)** IHC staining for the RAS downstream effector, p-Erk, on lungs obtained from mice described in C. In all figures, error bars represent the standard deviation of three different biological experiments done in triplicates. The statistical significance was measured by calculating the p values for all experiments comparing ITCH to EV, RAS/WT to ITCH or RAS; * indicates P value <0.05, **P value <0.01, ***P value <0.001.

After demonstrating the effects of ITCH on breast cell tumorigenicity *in vitro*, we set to test its effect on these cells *in vivo*. To prove that ITCH plays a role in breast tumorigenesis *in vivo*, we injected ITCH overexpressing MCF10A cells into the mammary fat pad (MFP) of Nod-SCID female mice. Two months later, we observed that ITCH expression in MCF10A cells didn't lead to tumor in these mice (data not shown) suggesting that ITCH alone is not enough to transform MCF10A cells. To have a proper model to study the role of ITCH *in vivo*, we overexpressed the different ITCH constructs mentioned above in H-RAS transformed MCF10A cells (MCF10A-RAS cells, [30]). First, we characterized the tumorigenic potential of these cells *in vitro*. We tested their survival and tumorigenicity, by colony formation and soft agar assays, respectively. We observed that more colonies were formed in wt ITCH overexpressing MCF10A-RAS cells, as compared to cells overexpressing either the WFPA or C830A mutant ITCH constructs, indicating a higher survival rate of these cells (Fig 3A). In a similar manner, soft agar assays clearly indicated that wt ITCH overexpression results in MCF10A-RAS cells having a growth advantage over control cells or cells over expressing WFPA or C830A ITCH (Fig 3B & S2A).

After characterizing these cells *in vitro*, we tested their tumorigenic potential *in vivo*. Cells were orthotopically injected into MFP of Nod-SCID mice and tumor formation was monitored. While no tumors were observed in MCF10A-ITCH cells, clones overexpressing both RAS and ITCH (RAS/ITCH) developed accelerated tumors, as early as one week post-injection. These mice had to be sacrificed one month after cell injection due to big tumors' formation. In comparison, cells over expressing RAS/ITCH-WFPA or RAS alone developed smaller tumors with slower growth rate (Fig 3C & S2B). Of note, mice injected with RAS/ITCH-C830A developed no tumors (Fig 3C). To confirm that ITCH enhances breast tumorigenesis by inhibiting the Hippo pathway, tumors were analyzed for YAP expression using immunohistochemistry (IHC). We observed strong nuclear staining of YAP in tumors obtained from cells overexpressing both RAS and wt ITCH, compared to cells overexpressing either RAS alone or RAS with ITCH-WFPA mutant (Fig 3D). To further decipher the role of ITCH on breast cancer progression into a metastatic disease, we examined lungs of these mice by staining for p-Erk, a downstream effector of RAS. While tumors obtained from cells overexpressing ITCH and RAS together showed a strong staining of p-Erk in large areas of the stained lung tissues (Fig 3E), indicating the presence of metastatic foci, tiny or even no metastatic foci were observed in animals bearing tumors of RAS and ITCH-WFPA or RAS alone, respectively (Fig 3E, F). Collectively, these data demonstrate that ITCH cooperates with RAS to accelerate breast tumor growth *in vivo*.

### ITCH knockdown inhibits the tumorigenic phenotype of MDA-MB435 breast cancer cells

**Figure 4:**
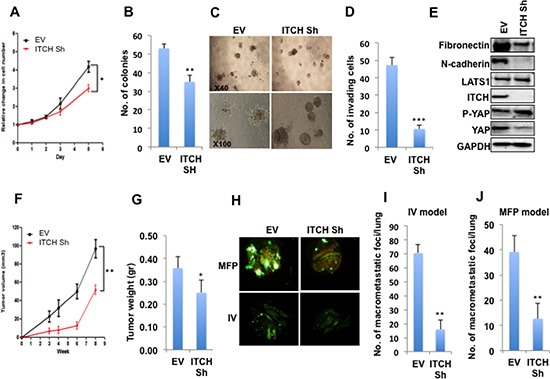
ITCH depletion inhibits MDA-MB435 breast cancer cell tumorigenic phenotypes **(A)** XTT proliferation assay showing the effect of ITCH knockdown on cell growth rate. **(B)** Quantification of cell survival rates using colony formation assay. **(C)** Micrographs of 3D cell culture of MDA-MB435 cells expressing either empty vector (EV) or ITCH Sh construct. **(D)** Quantitative presentation of Boyden Chamber Matrigel invasion assay results of MDA-MB435 cells expressing either EV or ITCH Sh construct. **(E)** Immunoblot analysis showing the effect of ITCH knockdown on EMT markers in MDA-MB435 cells. **(F)** Tumor growth curve of control and ITCH Sh MDA-MB435 cells injected in the MFP of Nod-SCID mice (n=6/group). Mice were analyzed 8-weeks post injection. **(G)** Graphical representation of tumor weight described in F. It was noted that tumors developed in control cells (EV) had a significant necrotic areas. **(H)** Representative micrographs of lungs obtained from Nod-SCID mice injected orthotopically into MFP or into the tail vein (IV) with control or ITCH Sh MDA-MB435 breast cancer cells constitutively expressing GFP. Mice were analyzed 6-weeks post injection. **(I)** Quantification of macrometastatic foci number obtained in the lungs of mice in the MFP model; (n=6/group). **(J)** Quantification of macrometastatic foci number obtained in the lungs of mice in the IV model; (n=6/group). In all figures, except those of mice, error bars represent the standard deviation of three different biological experiments done in triplicates. The statistical significance was measured by calculating the p values for all experiments related to EV; * indicates P value <0.05, **P value <0.01, ***P value <0.001.

Our results obtained from the overexpression system prompted us to test whether ITCH knockdown inhibits the tumorigenic phenotype of breast cancer cells. To this end we knocked down ITCH in the aggressive metastatic breast cancer cell line MDA-MB435 [31] using small hairpin (ShRNA) constructs expressed in lentiviral vectors. After infection and selection, stable clones were generated. To prove successful ITCH knockdown, we tested ITCH protein levels in either control cells or ITCH Sh clones using immunoblot analysis. As shown in Fig S3A, ITCH Sh clones showed a significant decrease in ITCH levels compared to control cells. To demonstrate the effect of ITCH knockdown on cell proliferation and survival of MDA-MB435 cells, we performed XTT and colony formation assays. As shown in Fig 4 A & B, both traits were attenuated by ITCH knockdown. To test whether ITCH knockdown can reverse the mesenchymal growth phenotype to a more epithelial one, we cultured ITCH Sh clones in 3D culture and tested their ability to form organized mammospheres. Indeed, ITCH Sh clones were able to form relatively more organized spheres compared to control cells (Fig 4C), which resembles a more epithelial phenotype. To further confirm that ITCH is a pro-invasive factor in breast cancer, we tested the effect of ITCH knockdown on the invasive potential of MDA-MB435 breast cancer cells using a Boyden chamber Matrigel invasion assay. Likewise, our results clearly demonstrated that ITCH depletion dramatically inhibits the invasion capability of MDA-MB435 cells (Fig 4D, S3B). To further dissect these phenotypes, we tested the effect of ITCH knockdown on levels of the mesenchymal markers N-Cadherin and fibronectin, using immunoblot analysis. Our results demonstrated that ITCH knockdown reduces N-cadherin and fibronectin protein levels in MDA-MB435 cells (Fig 4E & S1C).

### ITCH knockdown attenuates MDA-MB435 breast cancer cell metastatic potential *in vivo*

To further confirm that ITCH affects breast cancer cell tumorigenicity *in vivo*, ITCH-depleted MDA-MB435 cells, which constitutively express GFP, were orthotopically injected in MFP of Nod-SCID mice and rate of tumor growth was followed. We found that ITCH knockdown resulted in reduced tumor volume and weight (Fig 4F, G) indicating that ITCH knockdown is associated with reduced breast cancer cell tumorigenesis.

To support our *in vitro* findings that convincingly demonstrated that ITCH enhances the invasiveness of breast cancer cells, we decided to test ITCH depletion on seeding metastasis. To this end, we first injected GFP-labeled MDA-MB435 ITCH Sh and control cells in the tail vein of Nod-SCID mice and followed GFP dissemination in the internal organs of these mice, especially in the lungs. We observed that ITCH knockdown inhibited lung colonization compared to control cells (Fig 4H, lower panel &I). Since the IV model doesn't represent the whole metastatic cascade, we orthotopically injected the same cells in MFP of mice and followed metastatic foci development in the lungs of these mice. When comparing same size primary tumors, we found that ITCH knockdown resulted in reduced lung metastatic foci formation as compared to control cells (Fig 4H, upper panel & J). Moreover the number of mice that developed lung metastasis was less upon ITCH knockdown (Fig S3C). These results clearly demonstrate that ITCH depletion inhibits breast cancer metastasis *in vivo*.

### ITCH depletion inhibits metastatic traits of MDA-MB231 breast cancer cells

**Figure 5:**
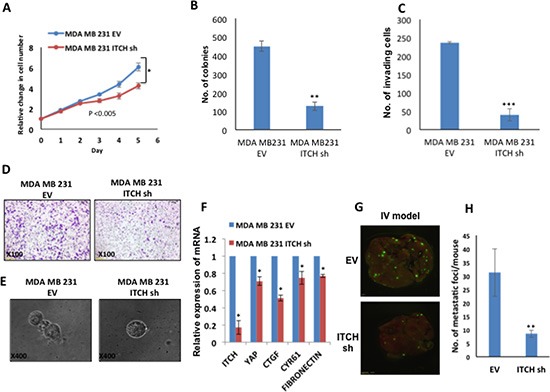
ITCH knockdown inhibits tumorigenicity of MDA-MB231 breast cancer cells **(A)** XTT proliferation assay showing the effect of ITCH knockdown on MDA-MB231 cell growth rate. **(B)** Quantification of cell survival rates using colony formation assay. **(C)** Quantitative presentation of Boyden Chamber Matrigel invasion assay results of MDA-MB231 cells expressing either empty vector (EV) or ITCH Sh construct. **(D)** Representative micrographs of Boyden Chamber Matrigel invasion assay of MDA-MB231 cells expressing either EV or ITCH Sh construct. **(E)** Micrographs of 3D cell culture of MDA-MB231 cells expressing either EV or ITCH Sh construct. **(F)** Real time PCR showing the effect of ITCH knockdown on YAP target genes. **(G)** Representative micrographs of lungs obtained from Nod-SCID mice injected into the tail vein (IV) with control or ITCH Sh MDA-MB231 breast cancer cells constitutively expressing GFP. Mice were analyzed 4-weeks post injection. **(H)** Graphical representation of the number of metastatic foci obtained in the lungs of mice in the IV model; *n=*6/group. In all figures, error bars represent the standard deviation of three different biological experiments done in triplicates. The statistical significance was measured by calculating the p values for all experiments related to EV. (*P value <0.05, **P value <0.01, ***P value <0.001).

To confirm that the observed phenotypes of ITCH depletion are not cell specific, we knocked down ITCH in the MDA-MB231 invasive breast cancer cells. Likewise, ITCH knockdown in this cell line, was associated with reduced cell proliferation and survival, as measured by XTT and colony formation assays, respectively (Fig 5A, B) similar to ITCH depleted MDA-MB435 cells. In addition, ITCH depletion in these cells dramatically inhibited their invasion capability (Fig 5C, D). Moreover, ITCH knockdown in MDA-MB231 cells inhibited the mesenchymal growth phenotype in 3D culture settings (Fig 5E). To explain whether these mesenchymal phenotypes are related to inactive Hippo pathway, we tested the effect of different ITCH knockdown on the expression level of YAP targets, CTGFβ, CYR61 and fibronectin using qRT-PCR. As shown in Figure 5F, the expression of these YAP targets is decreased upon ITCH knockdown. We further examine the effect of ITCH depeletion in these cells in vivo. GFP-labeled MDA-MB231 ITCH Sh and control cells were injected in the tail vein of Nod-SCID mice and GFP dissemination in the lungs was assessed. We found that ITCH knockdown attenuated lung colonization compared to control cells (Fig 5G, H). Altogether, these results clearly demonstrate that ITCH knockdown inhibits MDA-MB231 breast cancer cell growth, survival and invasion in vitro and metastasis in vivo further confirming that these effects are not cell specific.

### YAP knockdown rescues ITCH mediated phenotypes in MCF10A cells

**Figure 6:**
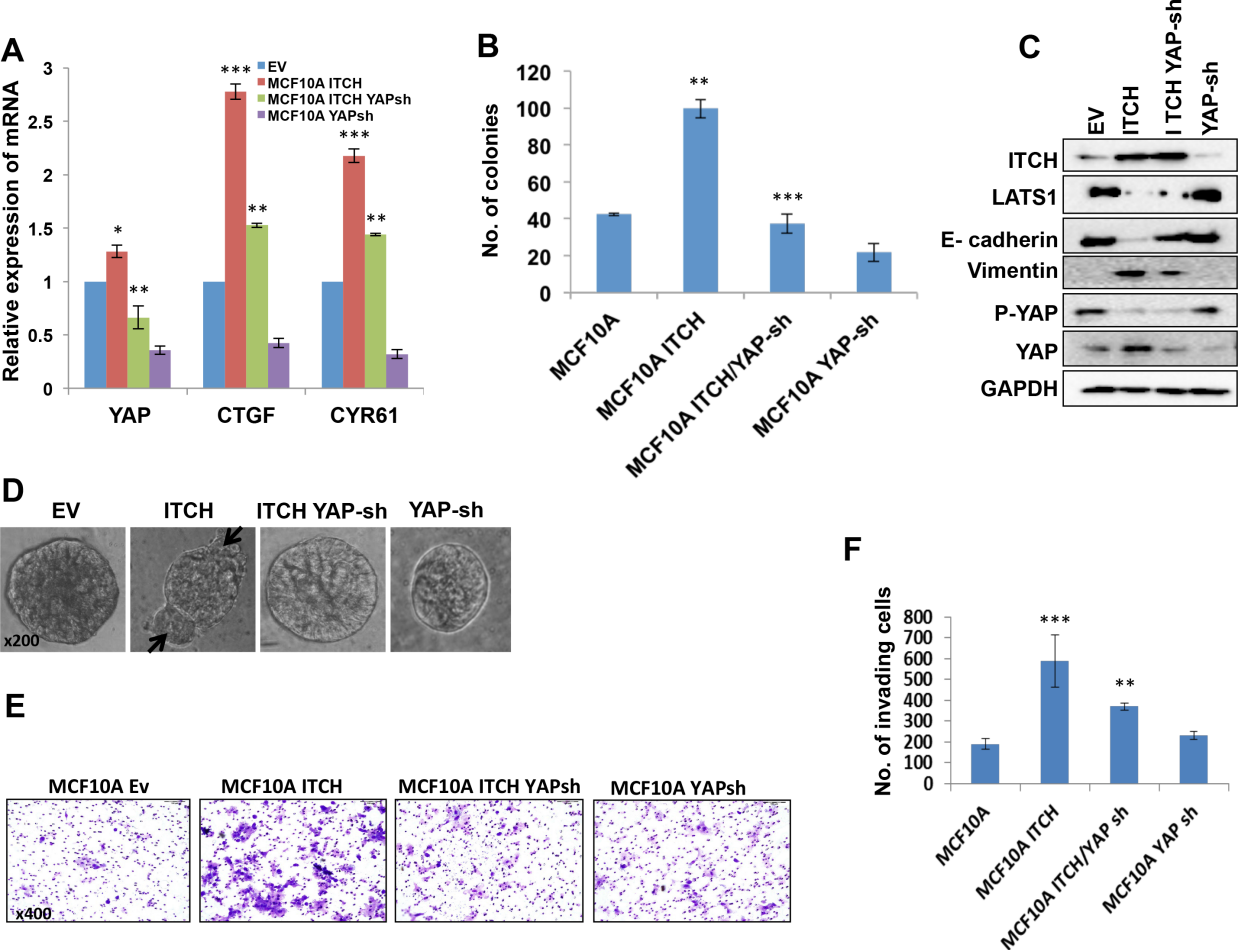
YAP knockdown rescues ITCH-mediated EMT phenotypes in MCF10A cells **(A)** qRT-PCR results showing successful YAP mRNA knockdown accompanied with downregulation of YAP target gene mRNA in 50% cell confluent culture. **(B)** Colony formation assay results showing the effect of YAP silencing on MCF10A cells overexpressing ITCH. **(C)** Immunoblot analysis showing the effect of YAP knockdown on EMT markers induced by ITCH overexpression. **(D)** 3D culture of MCF10A cells expressing either ITCH alone or ITCH and YAP Sh construct. Arrows point to abnormal structures in the mammospheres. **(E)** Representative pictures of the invading cells of the different indicated clones in Boyden chamber Matrigel invasion assy. **(F)** Quantification of Boyden chamber Matrigel invasion assay results in E. In all figures, error bars represent the standard deviation of three different biological experiments done in triplicates. The statistical significance was measured by calculating the p values for all experiments comparing ITCH to EV, ITCH-YAP-Sh to ITCH. * indicates P value <0.05, **P value <0.01, ***P value <0.001.

We have previously shown that ITCH enhances breast tumorigenesis by negative regulation of LATS1 [21], a key component of the Hippo pathway. This negative regulation of LATS1 was accompanied by reduced YAP activity. To further investigate the role of ITCH in breast carcinogenesis and to examine whether its function is mediated by activating YAP, we knocked down YAP in MCF10A-ITCH cells. To demonstrate a successful YAP knockdown we measured YAP mRNA levels using qRT-PCR. Our results indeed demonstrated reduced levels of YAP mRNA, indicating a successful YAP knockdown (Fig 6A). This reduction in YAP levels was accompanied by a downregulation of YAP target genes, *CTGFβ* and *CYR61*, confirming reduced YAP function as well (Fig 6A). To further confirm the effect of YAP knockdown on ITCH induced tumorigenicity in breast cells, we tested the effect of YAP knockdown on cell survival, using colony formation assay. While ITCH is capable of promoting cell survival, YAP knockdown was able to reverse this phenotype (Fig 6B).

To examine the consequences of YAP depletion in MCF10A-ITCH cells, we followed the expression of EMT markers by immunoblot analysis. We found that while ITCH overexpression leads to Vimentin upregulation and to E-cadherin downregulation, YAP knockdown displayed lower levels of Vimentin and higher levels of E-cadherin (Fig 6C & S1D). To demonstrate whether this effect on EMT related proteins would affect mammosphere culture of breast cells *in vitro*, we tested the effect of YAP knockdown on MCF10A-ITCH cell mammosphere formation. We observed that while overexpression of ITCH interferes with mammosphere formation and results in disorganized mammosphers characterized by abnormal bulging structures (Fig 6D second panel, arrows), YAP depletion was able to reverse the effect of ITCH on mammosphere formation to almost a normal and very well-circumscribed one (Fig 6D). To further investigate whether YAP knockdown is capable of reversing ITCH-induced invasion potential of breast cells, we tested the ability of YAP knockdown to reduce the effect of ITCH on MCF10A cell invasion in a Boyden chamber Matrigel invasion assay. As shown in Fig 6E & F, YAP knockdown was able to rescue the effect of ITCH on MCF10A invasiveness.

### YAP depletion attenuates ITCH-mediated tumorigenicity in MCF10A cells

**Figure 7:**
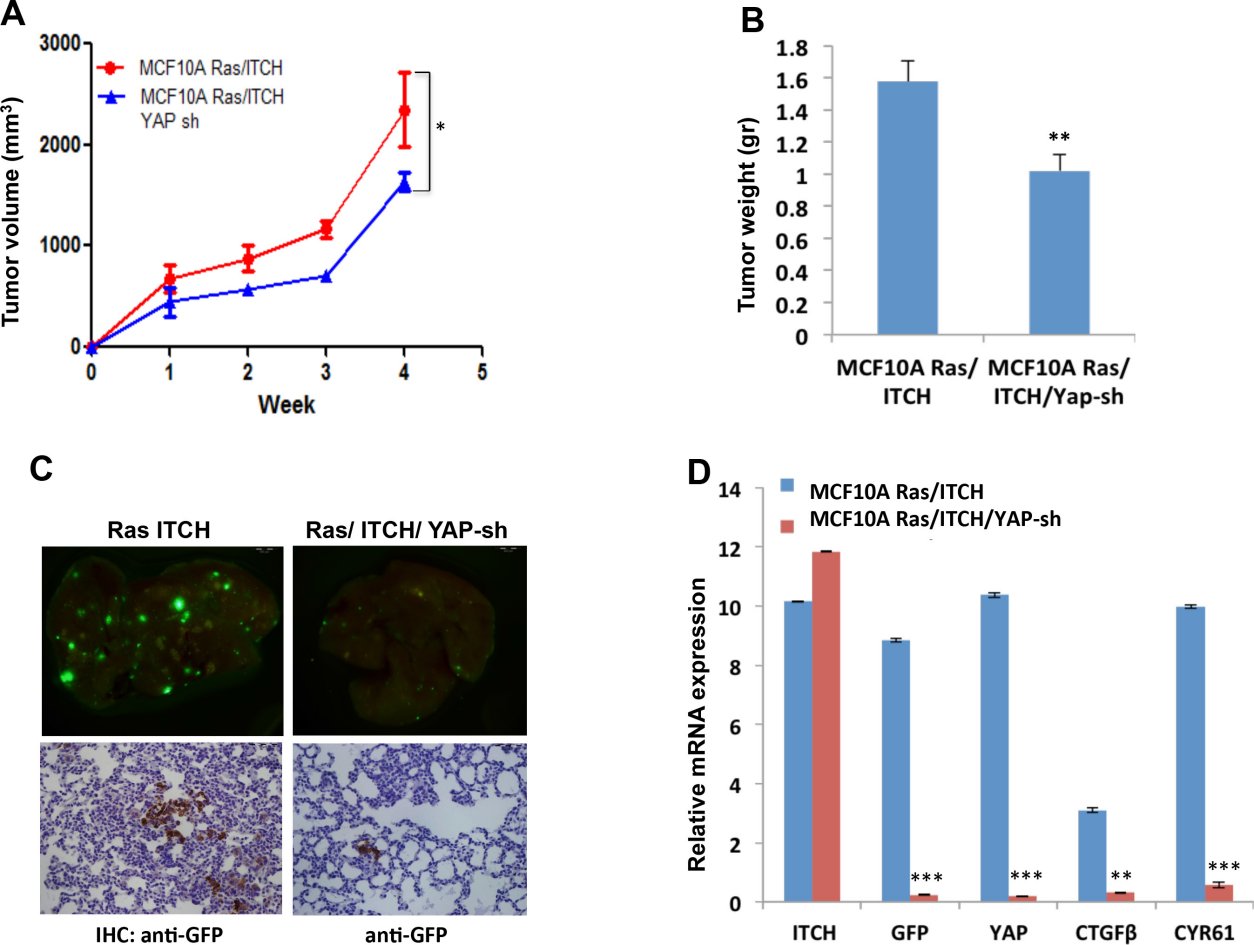
Effect of YAP knockdown on tumor progression in MCF10A cells overexpressing ITCH **(A)** Tumor growth rate curve showing the effect of YAP knockdown on tumor growth; (n=6mice/group). **(B)** Quantitative presentation of the effect of YAP knockdown on tumor volume. **(C)** Representative micrographs of lungs obtained from Nod-SCID mice injected orthotopically into MFP with ITCH overexpressing cells or cells overexpressing both ITCH and YAP Sh. Upper panels show metastatic foci of cells constitutively overexpress GFP while lower panels show IHC of lungs using anti-GFP. **(D)** Relative mRNA expression levels of the indicated genes on RNA obtained from lungs. In all figures, error bars represent the standard deviation of the same experiment made in triplicates. The statistical significance was measured by calculating the p values for all experiments comparing MCF10A Ras/ITCH to MCF10A Ras/ITCH/YAPsh. * indicates P value <0.05, **P value <0.01, ***P value <0.001.

To complement our *in vitro* findings demonstrating that ITCH induces tumor initiation and progression by activating YAP with an *in vivo* model, we injected GFP-labeled MCF10A-RAS/ITCH and MCF10A-RAS/ITCH/YAP-sh cells into MFP of Nod-SCID mice and followed tumor development and progression. We noticed that YAP knockdown in these cells leads to slower tumor development (Fig 7A), to ~40% reduction in tumor size (Fig 7B) and to less lung metastatic foci formation (Fig 7C), as assessed by GFP immunohistochemistry staining. To quantify the difference in metastatic foci formation, we measured the expression of GFP in lungs of mice from control and YAP Sh groups. Our qRT-PCR results indeed confirmed reduced GFP expression and levels of YAP targets upon YAP knockdown (Fig 7D). These data further confirm that ITCH induces its tumorigenic phenotype by deregulating the Hippo pathway and limiting its negative effect on its oncogenic effector, YAP.

### Increased ITCH protein levels correlate with metastatic breast cancer and worse survival

**Figure 8:**
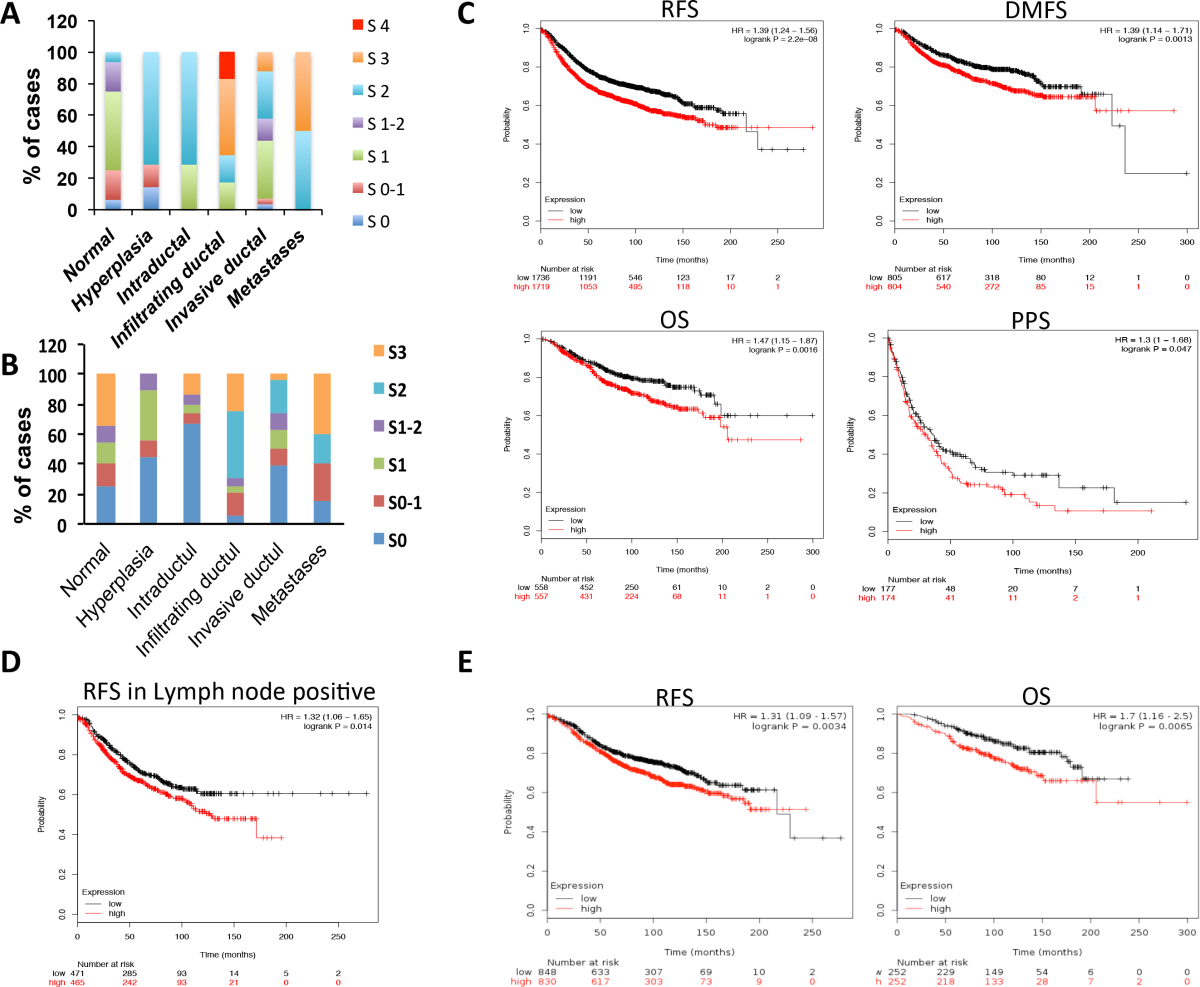
Prognostic significance of ITCH expression in different breast cancer subtypes **(A)** IHC scoring of ITCH protein in different breast cancer cases. The staining scores were as follows; 0= no staining, 1= weak staining, 2= moderate staining, 3= strong staining, 4= very strong staining. **(B)** IHC scoring of YAP protein in different breast cancer cases. The staining scores were as follows; 0= no staining, 1= weak staining, 2= moderate staining, 3= strong staining. C-E. Kaplan-Meier survival curves obtained from Kaplan-Meier Plotter recourse showing the correlation between ITCH mRNA expression and relapse-free survival (RFS), overall survival (OS), distant metastasis-free survival (DMFS), and post-progression survival (PPS) in breast cancer cohort (C); RFS in lymph node positive breast cancer samples (D); RFS and OS in Luminal A breast cancer patients (E).

To show the human relevance of ITCH expression in breast cancer, we stained tissue microarrays (TMAs) for ITCH and YAP using immunohistochemistry. Validation and specificity of the immunohistochemical staining is shown in Fig S4. Our data show that while low ITCH levels were detected in normal, hyperplastic and intraductal carcinoma tissue samples, high ITCH expression levels were seen in infiltrating or invasive ductal carcinoma samples as well as in samples that metastasized to secondary organs (Fig 8A). Representative images of the stained tissues are shown in Fig S5A. In fact, high expression of ITCH was observed in the majority (64%) of advanced stages of breast cancer cases (Fig S5B). As for YAP staining, normal and pre-neoplastic lesions showed variable expression (Fig 8B). Forty one percent of advanced stages of breast cancer, including infiltrating or invasive ductal carcinoma as well as metastasis, displayed high YAP levels (Fig S5B). Interestingly, we found a positive correlation between ITCH and YAP levels in about 70% of these samples (Fig S5C). To elucidate whether ITCH is expressed at equal frequency in the different breast cancer subtypes, we analyzed our TMA for the distribution of ITCH in ER+, HER2+, and triple-negative invasive breast cancers. However, no inverse correlation was found between ITCH expression and the distinct groups, likely due to the low number of cases in each category (data not shown).

To explore the prognostic value of ITCH expression in breast cancer, we evaluated mRNA expression of ITCH from publicly gene expression data set using the Kaplan-Meier Plotter resource [32]. Patients were divided into two groups (high and low ITCH expression) based on the Affymetrix 217094_s_at probe. These groups were then compared using the relapse-free survival (RFS), overall survival (OS), distant metastasis-free survival (DMFS), and post-progression survival (PPS). As shown in Fig 8C, Kaplan-Meier analysis revealed that high expression of ITCH was associated with shorter RFS (n=3455), shorter OS (n=1115), shorter DMFS (n=1609) and shorter PPS (n=351) in breast cancer patients. Interestingly, we also identified a significant association between high ITCH expression and shorter RFS in positive lymph node patients (n=936) (Fig 8D). Next, we tested the correlation between ITCH expression and it's prognostic value in the different breast cancer subtypes, basal, luminal A, luminal B and HER+. As shown in Fig 8E, Kaplan-Meier analysis revealed that high expression of ITCH is associated with shorter RFS (n=1678) and shorter OS (n=504) in luminal A breast cancer patients. No correlation was revealed in basal, Luminal B, and HER+ patients (data not shown). These data confirm that ITCH is overexpressed in metastatic breast cancer lesions and that its expression is associated with worse survival.

## DISCUSSION

In the current study we investigated the pro-tumorigenic function of ITCH in breast cancer. In particular, we demonstrate that ITCH enhances EMT through enhancing YAP oncogenic function. By contrast, a point mutation in the catalytic domain or WW1 domain of ITCH almost abolished its EMT-mediated effects. Furthermore, while overexpression of ITCH in breast cancer cells is associated with increased incidence of mammary tumor formation *in vivo*, its knockdown inhibited breast cancer cell tumorigenicity and reduced their metastatic potential, both, *in vitro* and *in vivo*. Mechanistically, we show that these ITCH-induced pro-tumorigenic phenotypes can be rescued, at least in part, by depleting YAP, further confirming that ITCH regulates the Hippo pathway. To demonstrate the human relevance of our findings, we tested ITCH expression in human breast cancer tissue samples and found that ITCH expression is significantly upregulated in invasive and metastatic breast cancer cases.

Our findings clearly indicate that ITCH positively regulates proliferation, survival and invasion of breast cancer cells. ITCH depletion in two metastatic breast cancer cells hampers their cell proliferation and survival, inhibits invasion ability and attenuated tumor growth and metastatic potential. These findings indicate that ITCH can regulate different steps of the metastatic cascade including early steps such as invasion (EMT) and later ones as survival and colonization (MET). Detailed characterization of ITCH-mediated phenotypes both in vitro, using Gelfoam cultures [33], and in vivo, using high quality GFP tumor and cellular imaging [33–37], shall be required to further decipher ITCH protumorigenic functions in breast cancer progression and metastasis.

Several studies have confirmed ITCH and other E3 ubiquitin ligases ability of regulating the Hippo pathway. For example, it has been recently shown that WWP1 and NEDD4 negatively regulate LATS1 by promoting its proteasomal degradation [18, 19]. Negative regulation of LATS1 function was shown to mediate WWP1-induced breast cancer cell proliferation [19]. It has also been demonstrated that NEDD4, NEDD4-2 and ITCH mediate polyubiquitination of AMOT/p130 [38], a member of AMOT family of proteins (AMOT/p130, AMOTL1 and AMOTL2) and resulted in regulating the downstream effectors of the Hippo pathway YAP and TAZ [15, 16, 39]. Intriguingly, ITCH appears to have dual antagonistic regulatory functions on the Hippo pathway. While we and others clearly demonstrated that ITCH negatively regulates the Hippo pathway by targeting LATS1 for degradation, Adler et al. [40] showed that AMOT/p130 directs ITCH towards the induction and inhibition of YAP1 and LATS1 degradation, respectively. Intriguingly when co-expressed together, ITCH and AMOT/p130 were shown to suppress cell growth, confirming an anti-tumor effect of ITCH. These findings might indicate that the effect of ITCH on the Hippo pathway is context dependent and that its E3 ligase function is tightly regulated. Future investigation will be required to uncover the role of ITCH activity in the different contexts.

Previously, we showed that ITCH interacts with LATS1 mainly through its first WW domain (WW1). Here we show that a point mutation in WW1 of ITCH is enough to abrogate ITCH induced pro-tumorigenic phenotypes *in vitro* and *in vivo*. Many WW domain-containing proteins have more than one WW domain and, usually, the functions of the different WW domains in the same protein are not redundant. Rather, WW domain-containing proteins may utilize alternative combinations of WW domains to bind different partners. For example, ITCH was recently shown to interact with AMOT/p130 via its WW1 and WW2 and not WW3 and WW4 [41]. Moreover, it has been shown that ITCH binds to p73α through its WW2 domain [42]. ITCH by itself can also be a target of WW domain interactions. Recently, we reported that ITCH, via its LPxY motifs, interacts with WW-domain containing protein, WWOX [43]. Interestingly, ITCH mediates K63-linked polyubiquitination of tumor suppressor WWOX that leads to WWOX nuclear localization and increased cell death. The choice between K48 and K63 seems to be another factor that dictates the effect of ITCH on its substrates and adds to the complexity of understanding the roles of ITCH in different cellular contexts. While K48-linkages are mostly associated with commitment for proteasomal degradation, K63-linked polyubiquitination plays established roles in DNA damage repair, protein kinase activation and trafficking [44]. Indeed, compelling evidence have recently shown that ITCH mediated K63-linked polyubiquitination of WWOX is associated with its novel function in the DNA damage response [45]. It is thus clear that ITCH could exert various functions depending on its substrate and on the stimuli that activate it.

YAP and TAZ are the most known downstream effectors of the Hippo pathway that execute and regulate the pathway responses to different cellular processes related to tumorigenesis [46]. Inactivation of the Hippo pathway and thus YAP hyperactivation results in higher proliferative rates in different tissues [6, 17, 47], enhanced cell survival and resistance to death induced by chemotherapy and anoikis [48–50], maintenance of a stem cell phenotype [51, 52] and EMT and metastasis [5, 49, 53]. Interestingly, our work demonstrate that the invasive properties induced by ITCH overexpression can be rescued by YAP depletion, indicating that, at least in part, ITCH inactivates the Hippo pathway function by increasing YAP function. These findings are in agreement with our previous findings demonstrating that ITCH overexpression leads to LATS1 degradation and less YAP phosphorylation and thus its hyperactivation [21]. Future work shall address whether LATS1-resistant mutants that are unable to be ubiquitinated by ITCH are protumorigenic.

ITCH is overexpressed in different cancer types [54] and in our present work, we show that ITCH is highly expressed in breast cancer samples especially in advanced metastatic cases and that these high levels correlate with high YAP expression levels, consistent with recent observations showing high YAP1 expression in multiple cancer subtypes [48, 55]. Furthermore, high ITCH mRNA has a prognostic value as it correlates with worse survival of luminal A breast cancer patients. These results indicate that ITCH might function as a pro-tumorigenic factor that supports a more aggressive type of breast carcinoma at least in part by modulating YAP function, the most downstream effector of the Hippo pathway.

## MATERIALS AND METHODS

### 

#### Cell culture and transient transfection

HEK293, MDA-MB435 and MDA-MB231 cells were grown in RPMI, supplemented with 10% fetal bovine serum (Gibco, NY), glutamine, and penicillin streptomycin (Beit Haemek, Israel). MCF-10A cells were grown in DMEM/F12 supplemented with 5% donor horse serum, 20 ng/ml epidermal growth factor (EGF), 10 μg/ml insulin, 0.5 μg/ml hydrocortisone, 100 ng/ml cholera toxin, and antibiotics. Overexpression of proteins was achieved by transient transfections using Mirus TransLTi (Mirus Bio LLC, Madison, WI).

#### Immunoblot Analysis

Cells were lysed by using Nonidet P-40 lysis buffer containing 50 mM Tris (pH 7.5),150 mM NaCl, 10% glycerol, 0.5% Nonidet P-40, and protease inhibitors. Western blotting was performed under standard conditions. Antibodies used were monoclonal anti-E. Cadherin and N. Cadherin (BD Biosciences, Lexington, KY), polyclonal anti-LATS1 (Bethyl, Montgomery, TX), phospho-YAPS127 and anti-YAP (Cell Signaling, Danvers, MA), Monoclonal anti-GAPDH (Calbiochem), polyclonal anti-fibronectin (Sigma Aldrich), monoclonal anti-Vimentin and monoclonal anti-ITCH (BD Biosciences, Lexington, KY).

### RNA extraction and reverse transcription-PCR and Real Time PCR

Total RNA was prepared using the TRI reagent (Sigma Aldrich) as described by the manufacturer. One microgram of RNA was used for cDNA synthesis using First-Strand cDNA Synthesis kit (Bio-Rad, Hercules, CA). Quantitative real-time PCR was performed using Power SYBR Green PCR Master Mix (Applied Biosystems, Foster City, CA). All measurements were performed in triplicate and standardized to the levels of GAPDH.

### In vivo tumorigenesis

1×10^6^ cells were suspended in 20μ l of 30% Matrigel cell culture medium and kept on ice until injection time. Cell suspension was then injected in the mammary fat pad of the abdominal mammary gland of Nod-SCID mice. Later, both primary tumors and other organs, including lungs, were excised for further analysis. For Intravenous injection, 1×10^6^ cells were suspended in 200μ l PBS and injected slowly into the tail vein. 8 weeks later animals were scarified, and lungs were excised and photographed with dissecting fluorescent microscope.

### Immunohistochemistry

Paraffin-embedded tissue sections or tissue microarray slides (US Biomax, Inc) were deparaffinized and rehydrated. Antigen retrieval was performed in 10 mM sodium citrate buffer pH 6.0 using pressurized chamber for 2.5 min. Endogenous peroxidase was blocked with 3% H_2_O_2_ for 10 min. The sections were then incubated with blocking solution (CAS Block, Invitrogen, Grand Island, NY) for 30 min to reduce non-specific binding followed by incubation with the primary antibody: monoclonal anti-ITCH antibody (Sigma Aldrich) [dilution of 1:100], anti-YAP (Epitomics) [dilution of 1:200], or anti-pErk (Cell signaling) [dilution of 1:100] in humidity chamber for overnight incubation. Slides were subsequently incubated with horseradish peroxidase-conjugated antibody for 30 min. The enzymatic reaction was detected in a freshly prepared 3,3 diamminobenzidine tetrahydrochloride using DAKO Liquid DAB Substrate-Chromogen (Carpinteria, CA) Solution for several minutes at room temperature. The sections were then counterstained with hematoxylin. Negative controls includes slides that were incubated with primary antibody alone without secondary antibody or slides that were incubated with secondary antibody alone without primary antibody. The staining scores were as follows; 0=No staining, 1=Weak staining, 2= Moderate staining, 3=Strong staining, 4=Very strong staining. Representative pictures of the negative and positive controls and the scoring system are shown in Fig S4 and S5A, respectively.

### Proliferation, Colony Formation and Matrigel invasion assays and 3D culture of MCF-10A cells

These tests were done as described previously [21].

### Statistical Analysis

Results were expressed as mean ± SD. Student t-test used to compare values of test and control samples. *P* < 0.05 indicated significant difference.

## SUPPLEMENTAL FIGURE LEGENDS


