# Supplementary material for: The ubiquitin E3 ligase ITCH enhances breast tumor progression by inhibiting the Hippo tumor suppressor pathway
Source: Oncotarget. 2014 Oct 11;5(21):10886–900. doi: 10.18632/oncotarget.2540 (PMC4279417; doi:10.18632/oncotarget.2540)
Supplement: Supplementary file 1 [file oncotarget-05-10886-s001.pdf]

SUPPLEMENTAL FIGURE LEGENDS

A

Densitometry Fig. 1A								
Cell type	EV		ITCH		WFPA		C830A	
percent	50	100	50	100	50	100	50	100
LATS1	1.0	0.63	0.37	0.68	1.05	1.1	1.05	0.94
p-YAP	1.0	0.72	0.5	0.33	0.55	0.78	1.1	1.0

C

Densitometry Fig. 4E		
Cell type	MDA MB 231 EV	MDA MB 231 ITCH sh
LATS1	1.0	1.57
P-YAP	1.0	2.0
YAP	1.0	0.25
ITCH	1.0	0.08
N-cadherin	1.0	0.09
Fibronectin	1.0	0.33

B

Densitometry Fig. 2E				
Cell type	EV	ITCH	WFPA	C830A
LATS1	1.0	0.33	0.62	0.63
P-YAP	1.0	0.2	1.0	0.8
YAP	1.0	4.66	1.0	1.33
ITCH	1.0	11	10	11
E-cadherin	1.0	0.0	1.125	0.75
Vimentin	1.0	9.0	0.0	0.0

D

Densitometry Fig. 6C				
Cell type	EV	ITCH	ITCH YAP sh	YAP sh
LATS1	1.0	0.11	0.22	1.0
P-YAP	1.0	0.125	0.125	0.75
YAP	1.0	3.0	0.75	0.25
ITCH	1.0	1.83	1.66	0.16
E-cadherin	1.0	0.09	0.63	0.81
Vimentin	0.0	1.4	0.8	0.1

Supplementry Figure S1: Densitometric analysis of immunoblots. Densitometric measurment of band intensity of Figures 1A, 2E, 4E and 6C.

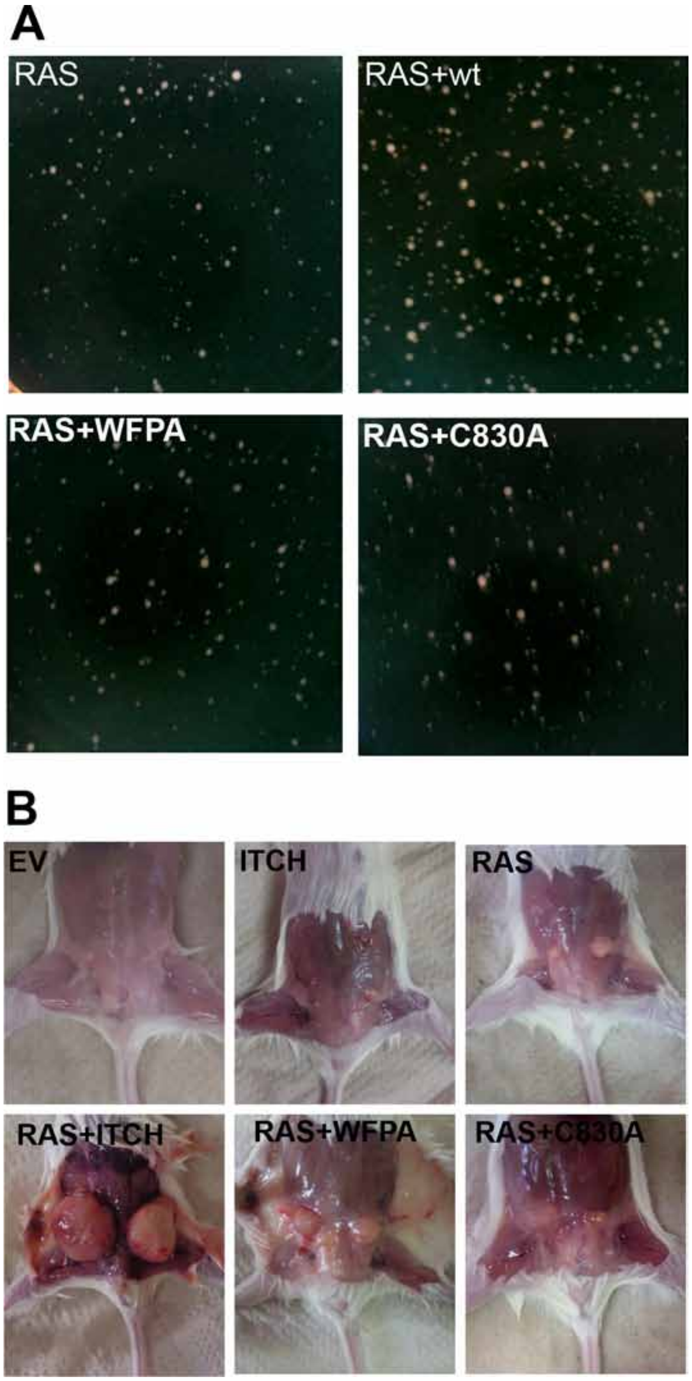

Supplementry Figure S2: ITCH enhances the tumorigenicity of H-RAS transformed MCF10A cells. (A) Representative micrographs of soft agar assay showing cell tumorigenic potential following the expression of the indicated proteins. **(B)** Representative micrographs of MFP tumors in Nod-SCID mice injected with MCF10A overexpressing the indicated proteins. Figure related to Fig 3.

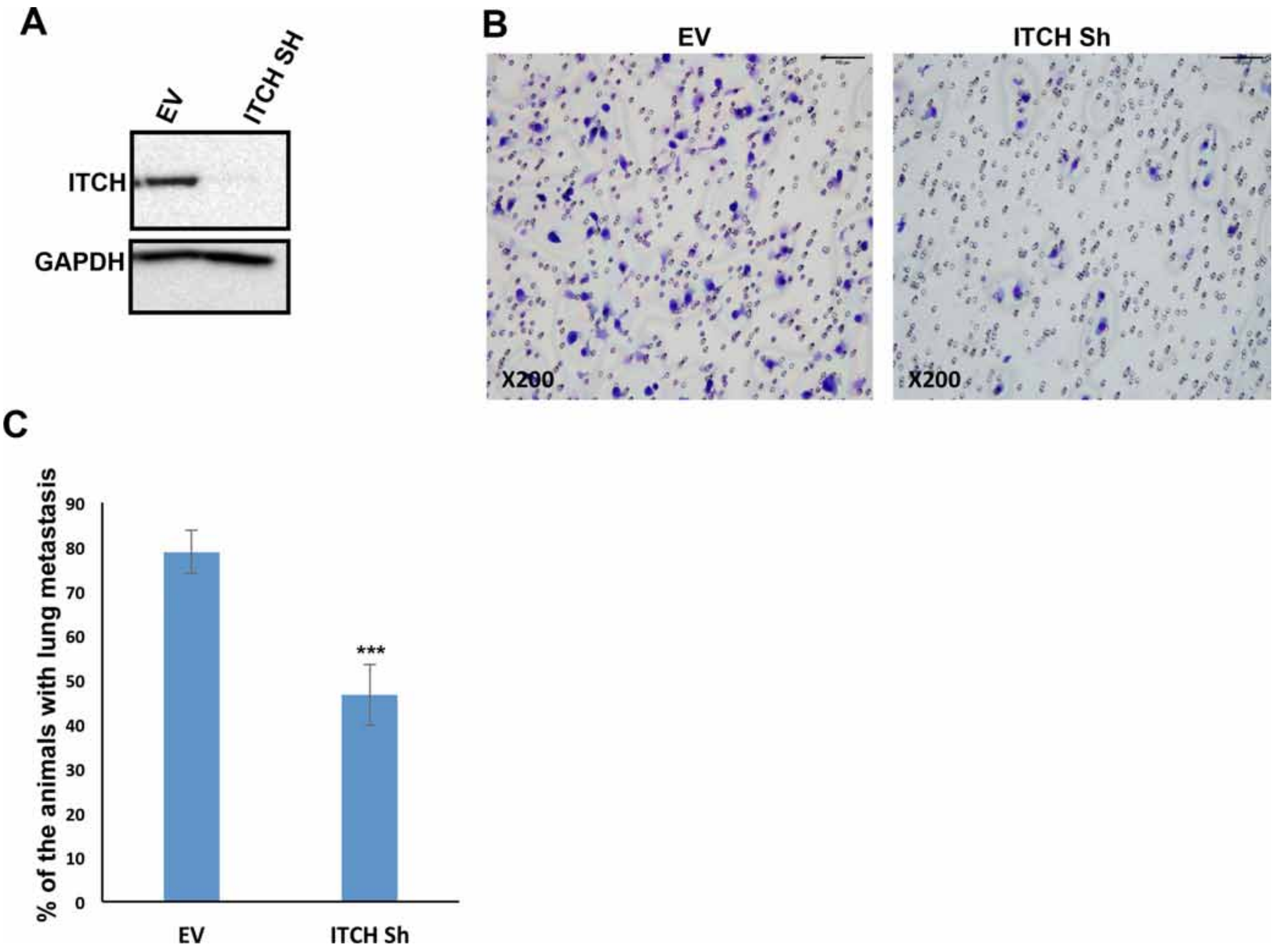

Supplementry Figure S3: ITCH knockdown inhibits breast cancer cell tumorigenic phenotype. **(A)** Immunoblot analysis showing successful ITCH knockdown in MDA-MB435 cells using ITCH ShRNA constructs. **(B)** Boyden Chamber Matrigel invasion assay results of MDA-MB435 cells expressing either empty vector (EV) or ITCH Sh construct. Figure related to Fig 4D. **(C)** Percentage of animals that developed lung metastatic foci in Fig 4H. *** indicates $P < 0.001$.

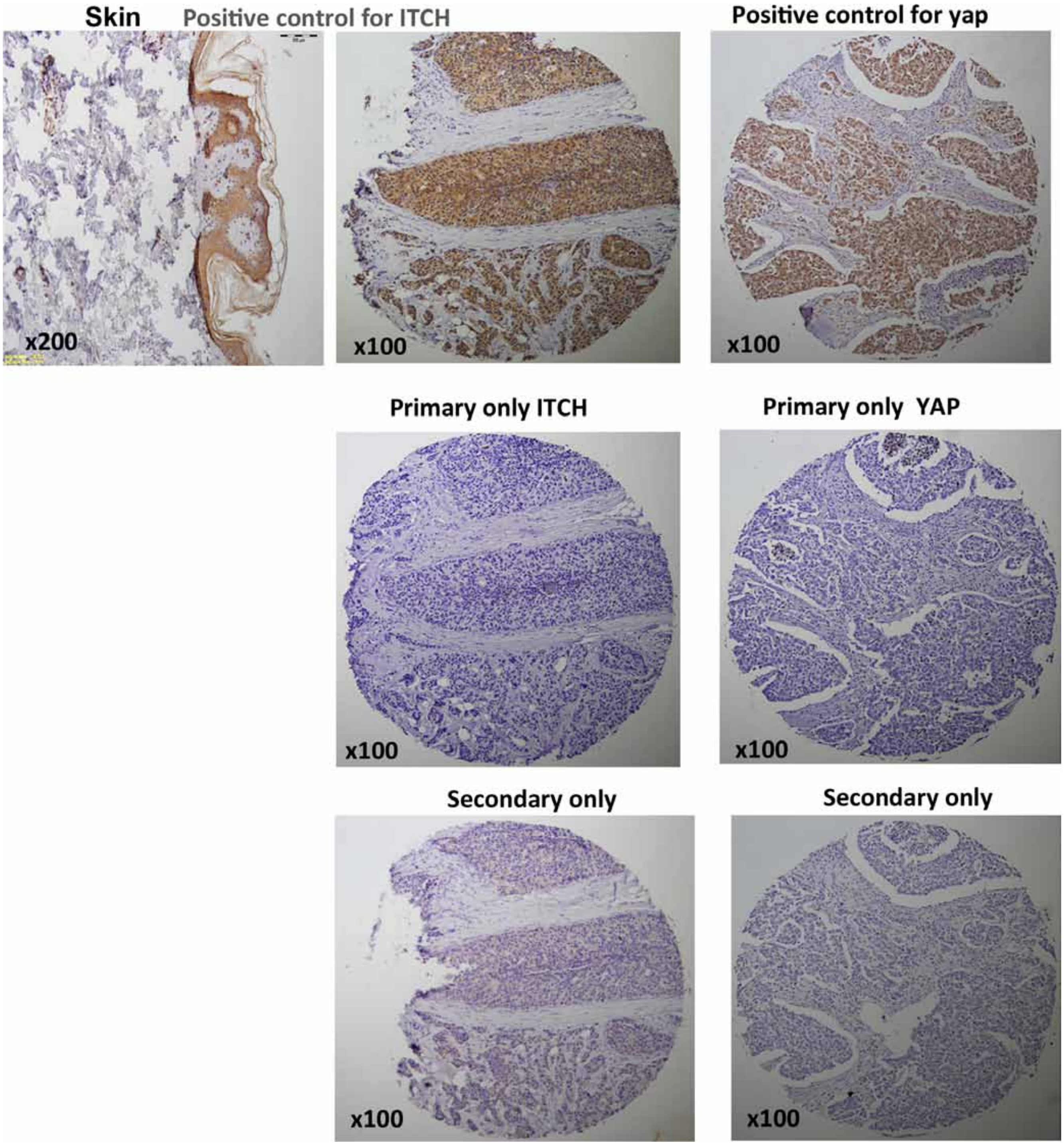

Supplementry Figure S4: Control slides for the IHC on TMA. Representative micrographs of positive and negative control slides used to validate the specificity of the immunohistochemical staining. Negative controls included slides stained with only either primary or secondary antibodies. Skin was included as an additional control for ITCH staining. Figure related to Fig 8A.

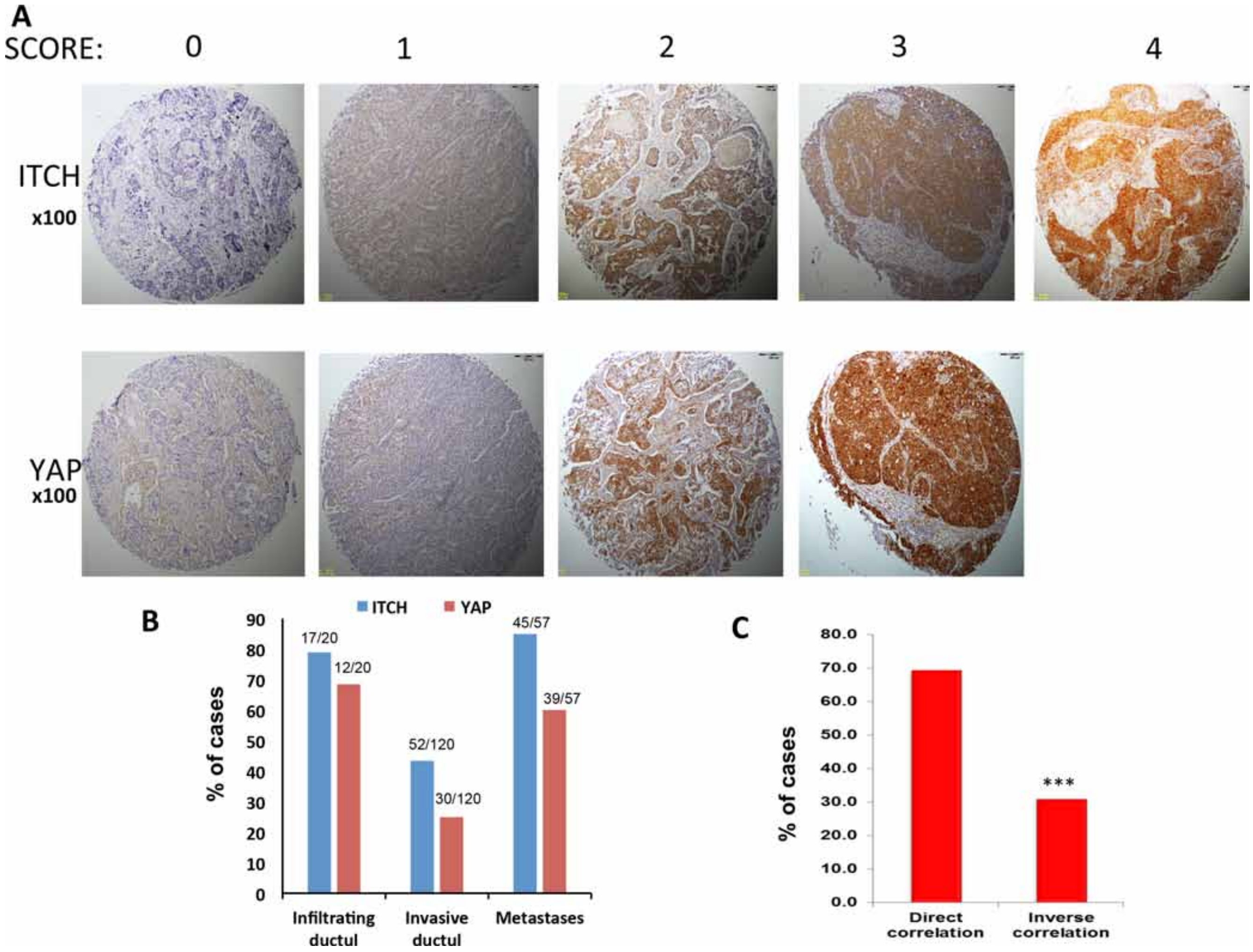

Supplementry Figure S5: Correlation between ITCH and YAP staining in breast cancer TMA. (A) Representative micrographs of breast cancer TMA stained, using IHC, for both ITCH and YAP proteins showing the scoring of the staining intensity. Percentage of advanced breast cancer cases including invasive or infiltrating and metastases displaying high levels of ITCH (scores 2, 3 or 4) and YAP (scores 2 and 3). Actual numbers are presented on top of each bar. **(C)** Statistical analysis of YAP immunohistochemical staining pattern in relation to ITCH staining in same samples. *** indicates $P<0.001$.
